# Comparison of sweep visual evoked potential of visual acuity and Snellen visual acuity in healthy and amblyopic children

**DOI:** 10.5935/0004-2749.2021-0130

**Published:** 2023

**Authors:** Murat Kasikci, Tuncay Kusbeci, Guliz Yavas, Onur Polat, Umit Inan

**Affiliations:** 1 Ophthalmology Department, Mugla Education and Research Hospital, Mugla, Turkey; 2 Ophthalmology Department, Izmir Bozyaka Education and Research Hospital, Izmir, Turkey; 3 Ophthalmology Department, Hacettepe University, Ankara, Turkey; 4 Ophthalmology Clinic, Dunyagoz Hospital, Bursa, Turkey; 5 Ophthalmology Clinic, Parkhayat Hospital, Afyonkarahisar, Turkey

**Keywords:** Amblyopia, Visual acuity, Visual evoked potentials, Vision tests, Humans, Child, Adolescent, Ambliopia, Acuidade visual, Potenciais evocados visuais, Testes visuais, Humanos, Criança, Adolescente

## Abstract

**Purpose:**

To evaluate the visual acuity of healthy and amblyopic children using sweep visual evoked potential and compare the results with those of Snellen visual acuity testing.

**Methods:**

A total of 160 children aged 6-17 years were included in the study. Of these, 104 (65%) were aged 7-17 years old, able to verbally communicate, and did not have any systemic or ocular pathology (Group 1). Group 2 included 56 (35%) children aged 6-17 years, able to verbally communicate, and had strabismus or anisometropic amblyopia whose best corrected visual acuity was between 0.1 and 0.8. All subjects underwent a detailed ophthalmological examination and sweep visual evoked potential measurement. Demographic characteristics, ocular findings, best corrected visual acuity, and sweep visual evoked potential results were recorded.

**Results:**

In Group 1, the mean and maximum visual acuity values for sweep visual evoked potential were lower than the Snellen best corrected visual acuity (p<0.001, for both, respectively). Bland-Altman analysis revealed that in Group 1, the distribution of the differences between the Snellen best corrected visual acuity and mean sweep visual evoked potential visual acuity was ±0.11 logMAR, and the distribution of the differences between the Snellen best corrected visual acuity and maximum sweep visual evoked potential visual acuity was ±0.023 logMAR. In Group 2, the mean and maximum sweep visual evoked potential visual acuity were lower than the Snellen best corrected visual acuity (p<0.001 and p=0.009, respectively). Bland-Altman analysis revealed that the distribution of the differences between the Snellen best corrected visual acuity and mean sweep visual evoked potential visual acuity was ±0.16 logMAR, and the distribution of the differences between the Snellen best corrected visual acuity and maximum sweep visual evoked potential visual acuity was ±0.19 logMAR.

**Conclusions:**

Sweep visual evoked potential visual acuity measurements have comparable results with Snellen visual acuity measurements. This technique is an objective and reliable method for evaluating visual acuity in children.

## Abstract (pt)

**Objetivo:**

Avaliar a acuidade visual através de po­tenciais evocados visuais de varredura em crianças saudáveis e ambliópicas, comparando-a com a acuidade visual pelo teste de Snellen.

**Métodos:**

Foram incluídas no estudo 160 crianças com idades entre 6 e 17 anos. Desse total, 104 crianças (65%) estavam entre 7 e 17 anos de idade, eram capazes de comunicação verbal e não tinham nenhuma patologia ocular ou sistêmica (Grupo 1). O grupo 2 incluiu 56 crianças verbais (35%) com idades entre 6 e 17 anos e portadoras de estrabismo ou ambliopia anisometrópica, com a melhor acuidade visual corrigida entre 0,1 e 0,8. Todos os pacientes foram submetidos a um exame oftalmológico detalhado e a uma medição do potencial evocado visual por varredura. Registraram-se as características demográficas, os achados oculares, a melhor acuidade visual corrigida e os resultados do potencial evocado visual por varredura.

**Resultados:**

No Grupo 1, os valores médios e máximos da acuidade visual pelo potencial evocado visual por varredura mostraram-se menores que a melhor acuidade visual corrigida medida através do teste de Snellen (p<0,001 para ambas as medições). Uma análise de Bland-Altman revelou que no grupo 1, a distribuição das diferenças entre a melhor acuidade visual corrigida pelo teste de Snellen e a média do potencial evocado visual por varredura foi de ± 0,11 logMAR, enquanto a distribuição das diferenças entre a melhor acuidade visual corrigida pelo teste de Snellen e o valor máximo do potencial evocado visual por varredura foi de ± 0,023 logMAR. No Grupo 2, os valores médio e máximo do potencial evocado visual por varredura mostraram-se menores que a melhor acuidade visual corrigida pelo teste de Snellen (respectivamente, p<0,001 e p=0,009). A análise de Bland-Altman revelou que a distribuição das diferenças entre a melhor acuidade visual corrigida pelo teste de Snellen e a média do potencial evocado visual por varredura foi de ± 0,16 logMAR, enquanto a distribuição das diferenças entre a melhor acuidade visual corrigida pelo teste de Snellen e o valor máximo do potencial evocado visual por varredura foi de ± 0,19 logMAR.

**Conclusões:**

As medidas da acuidade visual através do potencial evocado visual por varredura mostram resultados comparáveis às medidas da acuidade visual pelo teste de Snellen. Essa técnica é um método objetivo e confiável de se avaliar a acuidade visual em crianças.

## INTRODUCTION

Measuring the visual acuity (VA) is an essential part of ophthalmic examination. For verbal and cooperative patients, a subjective assessment is usually performed by using VA charts. A Snellen VA test is one of the most important methods used in clinics to evaluate VA. However, in infants and non-verbal or uncooperative patients, an objective assessment is required. Visually evoked potential (VEP), which is an electrophysiological technique, is one of the methods that can objectively evaluate visual function^(1)^. The most common stimulus used in a VEP test is a checkerboard pattern, which reverses every half-second. Alternatively, this pattern can also be made to appear (onset) and disappear (offset). The VEP recorded from the mid-occipital scalp is about 90% weighted, which means that it reflects the function of the central 10 degrees of the visual field and quantifies visual system function. Although previous studies have used pattern VEP to objectively assess VA, there is no consensus regarding the interpretation of VA assess­ments^(2,3)^, and the clinical usefulness of pattern VEP to determine VA is controversial^(4-6)^.

The sweep visual evoked potential (sVEP) is a new objective test for assessing VA and contrast sensitivity. It was first introduced and demonstrated by Regan for measuring refractive errors and further developed to rapidly assess VA and contrast sensitivity^(7)^. The sVEP is essentially the same as the steady-state pattern VEP except for the stimulus, which changes rapidly over time. For sVEP measurement, the stimulus is electronically swept in a spatial frequency over a particular range within a few seconds. It can be used to assess visual function in infants, young children, and people with special needs who have limited attention span and cannot participate in traditional subjective vision testing. However, the International Society for Clinical Electrophysiology of Vision (ISCEV) has not set standards for sVEP measurement.

In this study, we aimed to evaluate the VA of healthy and amblyopic children using sVEP and compare it with Snellen VA.

## METHODS

The current study included 160 children aged 6-17 years. Of these, 104 (65%) were aged 7-17 years old, able to verbally communicate, and did not have any systemic or ocular pathology (Group 1). Group 2 included 56 (35%) children aged 6-17 years, able to verbally communicate, and had strabismus or anisometropic amblyopia whose best corrected VA (BCVA, by Snellen measurements) were between 0.1 and 0.8. The study was approved by the Medical Ethical Committee of Afyon Kocatepe University Faculty of Medicine. All patients were informed about the study, and informed consent was obtained from their families.

All subjects underwent a detailed ophthalmological examination, and BCVA was obtained using a Snellen chart. Snellen VA measurements were then converted to logMAR^(8)^. Cyclopentolate (Sikloplejin 1%, Abdi İbrahim, Turkey) eyedrops were instilled to both eyes twice within 5 minutes, and cycloplegic refraction measurements were performed. Fundus examination was performed by slit-lamp biomicroscopy or indirect ophthalmoscopy. All children underwent sVEP measurement using the Metrovision-Vison Monitor™ (Metrovision, Monpack3, France) device. Patients with ocular pathologies, including pathologic myopia, cataract, glaucoma, and uveitis, history of intraocular or vitreoretinal surgery, and any systemic pathologies, such as Down syndrome or cerebral palsy, were excluded from the study.

### sVEP technique

sVEP was measured by the Metrovision-Vison Monitor™ (Metrovision, Monpack3, France) device. The test distance, which was based on the measured VA level, was 2 meters in Group 1 and 1.5 meters in Group 2. During the recording, the patients were asked to focus on the fixed red-colored point in the middle of the screen. Recordings were taken with standard silver chloride cupula electrodes in 2 channels via the Metrovision-Vision Monitor Bioelectric Recording Unit™ amplifier connected to an optoelectronic stimulator. The active electrode was located at a position above the inion equivalent to 10% of the distance between the inion (external occipital protuberance) and nasion (above the nose). The reference electrode was placed on the forehead, and the neutral electrode was placed in the earlobe.

**Figure 1:**
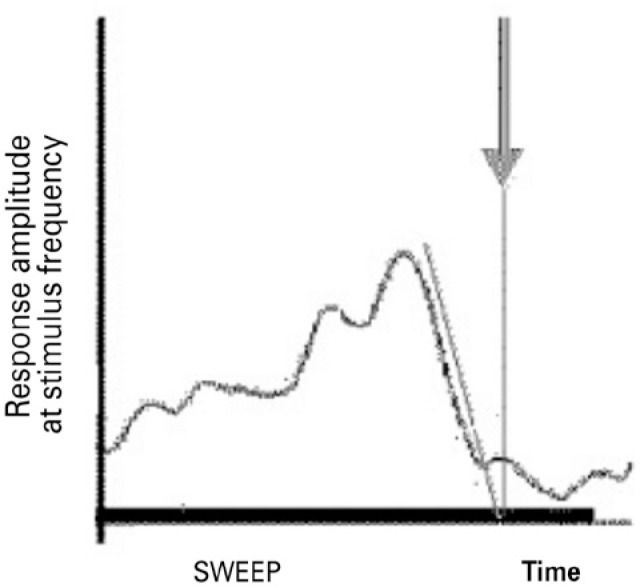
Each sweep is indicated by a vertical line, while its duration is indicated by a thick horizontal line. As the size of the pattern decreases, the amplitude of the response increases, reaches a maximum, then decreases rapidly. The sweep of the spatial resolution domain allows an estimation of visual acuity from the smallest pattern size producing a response.

The resolution of the optoelectronic stimulator was 1,024 × 768 pixels, and the average luminance was 50 cd/m^2^. The duration of sweep was 10 seconds; within 10 seconds, 20 different pattern sizes are presented in succession. The sVEP program generates a pattern stimulus that alternates at a high temporal frequency rate (in the range of 5-15 Hz), producing a steady-state visual evoked response (average: 12 Hz). A discrete Fourier transform was performed on the recorded signals and provided real-time measurements of the amplitudes and phases of the responses. This technique can detect a response extremely rapidly. To measure VA, the size of the pattern is rapidly reduced. This sweep of the spatial resolution domain allows VA estimation from the smallest pattern size that induces a response (Figure 1)^(9)^.

**Figure 2:**
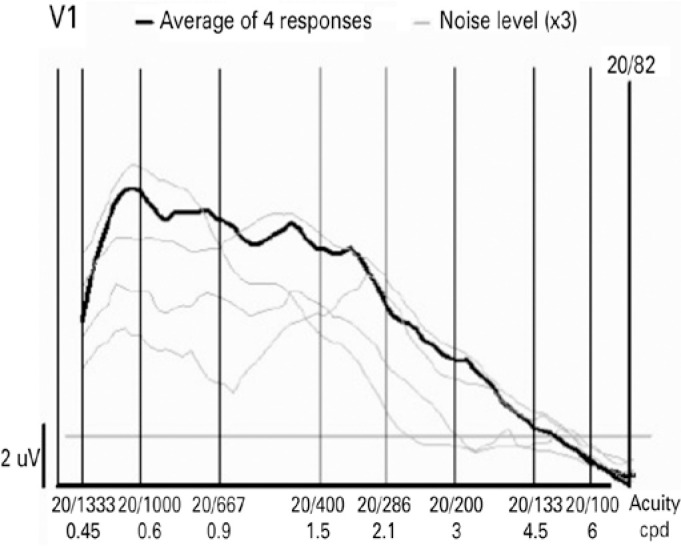
This graph shows the vector average of the different sweep responses recorded during the exam. From this vector average, the program automatically determines the visual acuity as the smallest pattern size that produces a response (20/82 in the present example).

Method to determine sVEP VA: Subjects had a test range VA of 0.08 (20/250)-0.81(20/25) in the sVEP program. The spatial frequency (SF) ranged from 2.5 to 24 cycles per degree and increased by 12% at each step. For each SF used as a stimulus, the VEP magnitude (µV) was plotted versus the SF. Typically, this SF tuning function drops to zero at finer SFs. Hence, selecting only those points on the final descending portion and performing linear regression on them allows the extrapolation of the straight line to 0 µV or to a noise “floor,” which is the point of intersection that defines the VEP SF limit (sVEP VA)^(10,11)^. The program calculates the vector average of the different sweep responses recorded during the exam. Vector averaging is an efficient way to reduce the noise level and to evaluate the reproducibility of responses. From this vector average, the program automatically determines the VA as the smallest size of pattern that induces a response (Figure 2). The mean and maximum VA measurements by sVEP were recorded as logMAR^(9-11)^.

Statistical analyses were performed by SPSS for Windows version 18.0 (SPSS Inc. Chicago, Illinois, USA). Data were further analyzed by paired t-test, Pearson correlation analysis, regression analysis, and Bland-Altman method. A p value <0.05 was considered statistically significant.

## RESULTS

**Figure 3:**
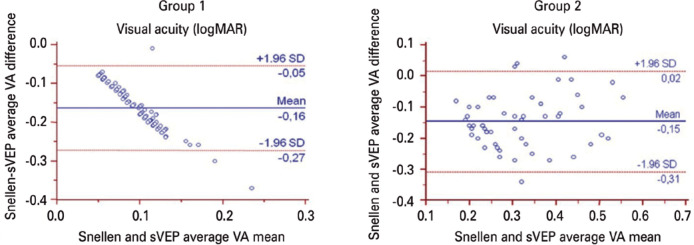
The Bland-Altman plot average difference was -0.16 logMAR in Group 1 and -0.15 logMAR in Group 2. The distribution of the differences between the Snellen best corrected visual acuity and mean sweep visual evoked potential visual acuity was ±0.11 logMAR in Group 1 and ±0.16 logMAR in Group 2. The 95% confidence intervals are represented by the dotted lines (±1.96).

**Figure 4:**
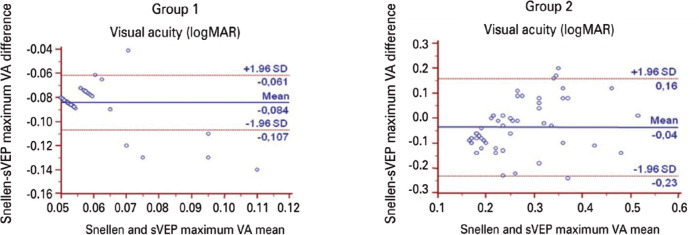
The Bland-Altman plot average difference was -0.08 logMAR in Group 1 and -0.04 logMAR in Group 2. The distribution of the differences between the Snellen best corrected visual acuity and mean sweep visual evoked potential visual acuity was ±0.023 logMAR in Group 1 and ±0.19 logMAR in Group 2. The 95% confidence intervals are represented by dotted lines (±1.96).

**Figure 5:**
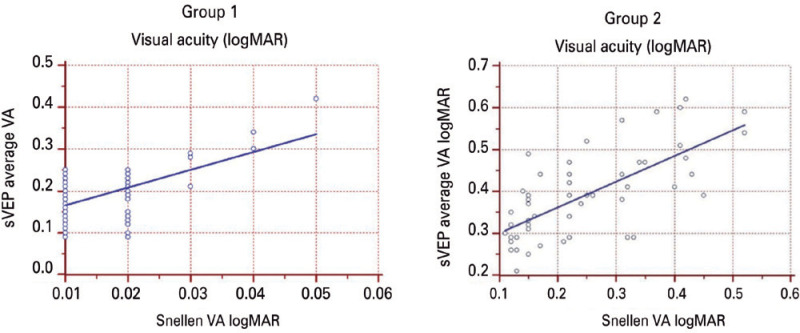
In Group 1, for the Snellen best corrected visual acuity and mean sweep visual evoked potential visual acuity, the regression equation was y=0.12 + 4.3x, and the coefficient of determination (R^2^) was 0.30. In Group 2, the regression equation was y=0.24 + 0.61x, and the R^2^ was 0.50.

**Figure 6:**
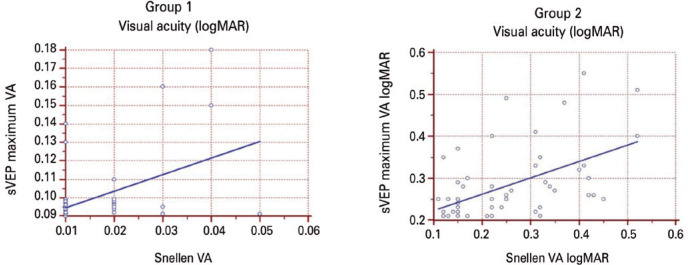
In Group 1, for the Snellen best corrected visual acuity and mean sweep visual evoked potential visual acuity, the regression equation was y=0.07 + 1.7x, and the coefficient of determination (R^2^) was 0.53. In Group 2, the regression equation was y=0.18 + 0.39x, and the R^2^ was 0.29.

Group 1 consisted of 57 (55%) men and 47 (45%) women. The mean age was 10.7 ± 3.2 years, and the mean spheric equivalent value was +0.78 ± 0.74 diopters. The mean BCVA measured by Snellen testing was 0.013 ± 0.007 logMAR, while the mean and maximum sVEP VA were 0.18 ± 0.057 logMAR and 0.10 ± 0.016 logMAR, respectively. The mean and maximum sVEP VA were significantly lower than the Snellen BCVA (p<0.001, for both, respectively). In this group, both the mean and maximum sVEP VA values correlated with the Snellen BCVA values (r=0.54, p<0.001; r=0.72, p<0.001, respectively). Bland-Altman plot analysis showed that the distribution of the differences between the Snellen BCVA and mean sVEP VA was ±0.11 logMAR, and the average acuity difference between the Snellen BCVA and mean sVEP VA was -0.16 logMAR (Figure 3). Meanwhile, the distribution of the differences between the Snellen BCVA and maximum sVEP were ±0.023 logMAR and -0.08, respectively (Figure 4). For the Snellen BCVA and mean sVEP VA, the regression equation was y=0.12 + 4.3x, and the coefficient of determination (R^2^) was 0.30 (Figure 5). For the Snellen BCVA and maximum sVEP VA, the regression equation was y=0.07 + 1.7x, and the R^2^ was 0.53 (Figure 6). In Group 1, the mean sVEP VA values correlated with the maximum sVEP VA values (r=0.63, p<0.001).

Group 2 consisted of 32 (57%) men and 24 (43%) women. The mean age was 9.7 ± 3.1 years, and the mean spheric equivalent value was +2.5 ± 2.3 D. In Group 2, the mean BCVA by Snellen test was 0.242 ± 0.114 logMAR, and the mean and mean maximum sVEP VA values were 0.39 ± 0.10 logMAR and 0.278 ± 0.08 logMAR, respectively. Similar to Group 1, the mean and maximum sVEP VA values were found to be significantly lower than the Snellen BCVA (p<0.001 and p=0.009, respectively). In this group, both the mean and maximum sVEP VA values also correlated with the Snellen BCVA values (r=0.71, p<0.001; r=0.54, p<0.001, respectively). Bland-Altman plot analysis showed that the distribution of the differences between the Snellen BCVA and mean sVEP VA was ±0.16 logMAR, and the average acuity between Snellen BCVA and mean sVEP VA was -0.15 logMAR (Figure 3). Meanwhile, the distribution of the differences between the Snellen BCVA and maximum sVEP were ±0.19 logMAR and -0.04 logMAR, respectively (Figure 4). For the Snellen BCVA and mean sVEP VA, the regression equation was y=0.24 + 0.61x, and the R^2^ was 0.50 (Figure 5). For the Snellen BCVA and maximum sVEP VA, the regression equation was y=0.18 + 0.39x, and the coefficient of R^2^ was 0.29 (Figure 6). In Group 2, the mean sVEP VA values correlated with the maximum sVEP VA values (r=0.73, p<0.001).

## DISCUSSION

In this study, we found that in healthy children, the sVEP VA values were 0.45 octaves lower than the Snellen BCVA values. Furthermore, the sVEP VA values of strabismic or anisometropic amblyopic children were 0.30 octaves lower than the Snellen BCVA values.

The parameters of sVEP, such as screen luminance, temporal frequency sweep type, sweep range, and direction of sweep affect the resulting VA measurements. Linear or logarithmic sweep types are used in an sVEP test^(12,13)^, and Tyler et al.^(12)^ have suggested the use of a linear sweep for VA measurements. In another study, a logarithmic sweep instead of a linear sweep has been suggested^(13)^. The authors used checkerboard stimuli that were swept logarithmic steps to measure the sVEP VA, and they reported that the sVEP VA highly correlated with the subjective VA of subjects with a normal VA as well as with the reduced VA of subjects with ocular pathologies^(13)^. In this study, we used checkerboard pattern stimuli that were swept logarithmic steps. Regarding linear or logarithmic sweep types, it must also be considered whether the sweep is continuous or sampled. A sampled sweep consists of a number of contrasts or SF gratings presented during the fixation period in a VEP recording^(13)^. In our recording system, a sampled sweep was used.

Various luminance levels have been used in sVEP studies (between 40 and 220 cd/m^2^). Allen et al.^(14)^ found that the VA increases with luminescence in both infants and adults. In addition, the increased luminosity and sharpness are shallower and less pronounced in babies. In our study, the luminance level was 50 cd/m^2^, and the resolution of the optoelectronic stimulator was 1,024 × 768 pixels. In their study, Good and Hou used luminance levels of 109 cd/m^2^ and 20 cd/m^2^ in normal children between 7 months and 4 years of age. These two luminance values were similarly terminated by the sVEP line sharpness; in children with cortical visual loss, sharpness values are better at low luminances^(15)^.

The direction of the contrast sweep can change the measured threshold value. When downsweeps are used to measure contrast sensitivity, an adaptation to the original high contrast may occur, increasing the threshold value. For this reason, upward sweepers are used for contrast threshold measurement. Another parameter that affects the sVEP threshold is the electrode location. The sVEP is not included in the ISCEV standards for VEP records. However, the active, ground, and reference electrodes are usually placed at locations based on the ISCEV standards^(16)^.

The validity and reliability of the sVEP is supported by several clinical trials. It has been proven throughout the studies that the sVEP test is a valid and reliable method for measuring VA in various age groups. Norcia and Tyler^(17)^ used two different temporal frequencies for VA in infants (6 and 10 Hz). They found good reproducibility for both temporal frequencies in the context of the highest sharpness values. The researchers also suggested that sVEP test had better VA and contrast sensitivity in a group of patients than in individual patients. Hamer et al. showed that sVEP studies can capture slight differences between the eyes, have better reproducibility than behavioral tests, and are sensitive when assessing vision loss in children^(18)^. The researchers also suggested that the sVEP test had better VA and contrast sensitivity in a group of patients than individual patients^(18)^.

Previous studies have reported good correlation between the sVEP test and different VA measurement methods. Sokol et al.^(19)^ compared preferential looking (PL) acuity (for stationary and for phase alternating gratings) with sVEP acuity in a group of infants between the ages of 2 and 10 months. They found that sVEP acuity was 1.5-2.5 octaves higher than the PL acuity for stationary gratings (a 1-octave difference is a doubling or halving of the number of cycles per degree). Arai et al.^(3)^ evaluated 100 patients with ocular pathologies by Snellen VA and sweep pattern reversal VEP and reported a correlation between these two methods. Furthermore, Katsumi et al.^(20)^ found a good correlation between PL acuity and sVEP acuity in children with various ocular diseases. They found that the sVEP values were lower and higher in cases where the PL acuity was better or worse than 6/38, respectively. In another study, Ridder et al.^(21)^ reported that VA estimation by sVEP that used stimuli with horizontal gratings before amblyopia treatment was a good predictor for the VA after treatment. Additionally, Da Costa et al.^(22)^ evaluated the VA of 37 patients with spastic cerebral palsy using sVEP and behavioral methods, and they determined that electrophysiological methods are more effective and dependable that motor dysfunctions in these individuals may affect the measurement methods.

Wizov et al.^(23)^ compared the results of sVEP and recognition acuity measurements in children with organic diseases, nystagmus, strabismus, and congenital ptosis, and they found a high correlation between sVEP and recognition acuity in children with organic diseases and strabismus with alternans fixation; however, the correlation in children with strabismus was low. In this study, we also found a high correlation between the mean and maximum sVEP VA and Snellen VA in children with visual impairment due to strabismus or anisometropia.

In conclusion, sVEP VA measurements are comparable with Snellen VA measurements. sVEP is an objective and reliable method for evaluating VA in both healthy and amblyopic children.
